# Obesity and the poor women living in urban slum areas: health system response

**DOI:** 10.1186/1471-2458-12-S2-A12

**Published:** 2012-11-27

**Authors:** Digna N Purwaningrum, Mubasysyir Hasanbasri, Laksono Trisnantoro

**Affiliations:** 1 Center for Health Service Management, Faculty of Medicine, Gadjah Mada University, Jogjakarta, Indonesia; 2 Postgraduate Program on Health Policy and Health Service Management, Faculty of Medicine, Gadjah Mada University, Jogjakarta, Indonesia

## Background

Obesity is one of the risk factors for non-communicable disease (NCD). The prevalence of overweight and obesity among the poor in Indonesia is increasing. Indonesia is also experiencing an epidemiological transition where the trend for causes of death is changing from infectious disease to NCD. Following previous studies on obesity among the poor, we study the risk factors for obesity in urban slum areas in order to seek for the appropriate health system solution to this matter.

## Materials and methods

We used a case-control design with 70 cases of obese women and 70 controls of non-obese women (WHO-IASO-IOTF obesity criterion for Asian population was used to determine the obesity status). In-depth interviews were conducted to 18 women respondent from three different slum areas in Yogyakarta city. We performed data triangulation for qualitative data. This study was conducted in April - October 2011.

## Results

The major risk factors for obesity among poor women living in urban slum areas were partly due to low level of physical activity (OR 8.0) and excessive carbohydrate intake (OR 1.5). The respondents stated that they couldn’t buy the healthy food because its price is higher than carbohydrate-based food. This study documented the lack of information about affordable and healthy food being discussed by community health worker and health educator. From the study findings, the expected response from the health system was the government needs to improve awareness and promotion of healthy living especially within vulnerable population.

## Conclusion

Poor nutrition is not the only source of hunger among the poor, but also obesity. This finding highlights the importance of collaboration between public health centers and community groups in the slum area in order to create awareness of the obesity problem. Public policies that focus on urban poor population need to be improved.

